# Possible Involvement of DNA Methylation in TSC1 Gene Expression in Neuroprotection Induced by Hypoxic Preconditioning

**DOI:** 10.1155/2022/9306097

**Published:** 2022-09-09

**Authors:** Ruifang Qi, Yabin Xie, Xiaolu Zhang, Shuyuan Jiang, Xiaolei Liu, Wei Xie, Xiaoe Jia, Rengui Bade, You Liu, Kerui Gong, Wenjie Yang, Guanghui Guo, Kai Sun, Chunyang Zhang, Ruijuan Han, Guo Shao

**Affiliations:** ^1^Department of Basic Medicine and Forensic Medicine, Baotou Medical College of Inner Mongolia University of Science and Technology, Baotou, China; ^2^Inner Mongolia Key Laboratory of Hypoxic Translational Medicine, Baotou Medical College, Baotou, China; ^3^Beijing Key Laboratory of Hypoxic Conditioning Translational Medicine, Xuanwu Hospital, Capital Medical University, Beijing, China; ^4^Department of Neurosurgery, The First Affiliated Hospital of Baotou Medical College, Inner Mongolia, China; ^5^Department of Oral and Maxillofacial Surgery, University of California san Francisco, San Francisco, USA; ^6^Center for Translational Medicine and Department of Laboratory Medicine, The Third People's Hospital of Longgang District, Shenzhen, China; ^7^Department of Cardiology, The People's Hospital of Longgang District, Shenzhen, China

## Abstract

**Background:**

It has been reported that ischemia and ischemic preconditioning (IPC) have different effects on the expression of tuberous sclerosis complex 1 (TSC1), which may contribute to the tolerance to ischemia/hypoxia with the increase of autophagy. The mechanisms of TSC1 differential expression are still unclear under ischemia/IPC conditions in hippocampal Cornu Ammon 1 (CA1) and Cornu Ammon 3 (CA3) area neuronal cells. While we have shown that 5-Aza-CdR, a DNA methyltransferase inhibitor, can upregulate TSC1 and increase hypoxic tolerance by autophagy in vivo and in vitro, in this study, we examined whether DNA methylation was involved in the differential expression of TSC1 in the CA1 and CA3 regions induced by hypoxic preconditioning (HPC).

**Methods:**

Level of rapamycin (mTOR) autophagy, a downstream molecular pathway of TSC1/TSC2 complex, was detected in HPC mouse hippocampal CA1 and CA3 areas as well as in the HPC model of mouse hippocampal HT22 cells. DNA methylation level of TSC1 promoter (-720 bp~ -360 bp) was determined in CA1 and CA3 areas by bisulfite-modified DNA sequencing (BMDS). At the same time, autophagy was detected in HT22 cells transfected with GFP-LC3 plasmid. The role of TSC1 in neuroprotection was measured by cell viability and apoptosis, and the role of TSC1 in metabolism was checked by ATP assay and ROS assay in HT22 cells that overexpressed/knocked down TSC1.

**Results:**

HPC upregulated the expression of TSC1, downregulated the level of P-mTOR (Ser2448) and P-p70S6K (Thr389), and enhanced the activity of autophagy in both in vivo and in vitro. The increased expression of TSC1 in HPC may depend on its DNA hypomethylation in the promoter region in vivo. HPC also could reduce energy consumption in HT22 cells. Overexpression and knockdown of TSC1 can affect cell viability, cell apoptosis, and metabolism in HT22 cells exposed to hypoxia.

**Conclusion:**

TSC1 expression induced by HPC may relate to the downregulation of its DNA methylation level with the increase of autophagy and the decrease of energy demand.

## 1. Introduction

The brain is the most sensitive to hypoxic/ischemic injury due to its high demand for oxygen and glucose [1, 2]. The hippocampal CA1 area is sensitive to hypoxic/ischemic injury extremely, while its adjacent CA3 area is remarkably resistant to hypoxic/ischemic injury [3, 4]. It has been proposed that selective activation of molecules, such as HSP70 and phospholipase A2, contributes to the differential vulnerability of CA1 versus CA3 [5, 6]. Papadakis et al. found that tuberous sclerosis complex 1 (TSC1), an upstream regulator of mTOR, played a role in the hypoxic/ischemic tolerance of hippocampal CA1 and CA3 area neuronal cells after ischemia/IPC treatment [7]. Thus, it should be essential to elucidate the expression mechanism of TSC1, to understand the differential ischemic/hypoxic vulnerability of CA1 and CA3 and to clear the endogenous neuroprotective mechanism induced by ischemic/hypoxic preconditioning (I/HPC).

I/HPC, an endogenous cellular protection mechanism, refers that sublethal ischemic/hypoxic exposure can increase the resistance of cells, tissues, or organs to subsequent lethal stimulation [8, 9]. Downregulation of DNA methyltransferases (DNMTs), enzymes regulating DNA methylation levels, was proposed to be involved in neuroprotection as a kind of endogenous cellular mechanism induced by HPC [10]. DNA methylation level associated with gene expression and hyper- and hypomethylation means low and high gene expression levels [11]. We had shown that the expression of TSC1 was upregulated, and the TSC1-autophagy signaling pathway was activated by DNMT inhibitor 5-Aza-CdR [12]. Thus, the differential expression of TSC1 in CA1 and CA3 areas induced by I/HPC may be due to the change of DNA methylation level, and the TSC/mTOR/autophagy pathway may be involved in the endogenous neuroprotective mechanism.

Autophagy is an important process to maintain the metabolism essential for cell survival under stress situations, including starvation and hypoxia [13]. Autophagy has been revealed to be critical in the regulation of energy balance in the brain [14, 15]. Decreasing energy consumption and increasing energy supplements have been suggested to be vital mechanisms in neuroprotection under hypoxia conditions [16]. In this study, we hoped to investigate the effect of HPC on the DNA methylation level and the expression of TSC1 on the activity of the TSC/mTOR/autophagy pathway, and on energy metabolism in the CA1 and CA3 regions using HPC mouse models. To prove the neuroprotective role of TSC1 in hypoxia tolerance further, cell viability, apoptosis, and energy consumption were detected in mouse hippocampal HT22 cells overexpressing/knocking out TSC1.

## 2. Materials and Methods

### 2.1. Animal Model

**Figure 1:**
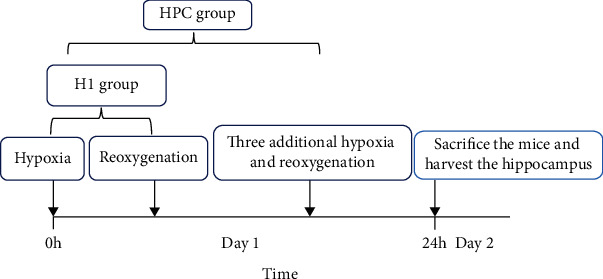
The flow chart of animal experiment.

A total of 153 male adult ICR mice, weighing 18-22 g, were obtained from Beijing Vital River Laboratory Animal Technology Co. Ltd. The animal room we used was kept at a constant temperature of 20°C. The mice were placed into a 125 mL jar with fresh air, which was sealed with a rubber plug. The animals were removed from the jar as soon as gasping appeared and switched to a second similar fresh air jar which was immediately sealed again. The time between the beginning of airtightness and the appearance of the first gasping was termed “tolerance time” or “hypoxia tolerance” for each run. This procedure was repeated for four times (HPC group), one time (H1 group), or zero time (control group) (Figure 1). The tolerance limit point is judged by the phenomenon of “gasping” of animals [17]. This study followed the US National Institutes of Health principles for laboratory animal care, and the protocol was approved by the Committee on the Ethics of Animal Experiments of the Baotou Medical College (permission number: BTMC20180302-001).

### 2.2. Cell Model

HT22 cells were maintained in Dulbecco's modified Eagle's medium (DMEM) (Gibco, Grand Island, NY, USA) as previously described [18]. The condition of the HPC group was 1% O_2_/94% N_2_/5% CO_2_ after 30 minutes of incubation and was changed to normal oxygen (21% O_2_/74% N_2_/5% CO_2_) for 30 minutes of incubation, so the cycle was alternately carried out for 4 times. Then, the cells were cultured together with the H1 group cells under hypoxic conditions of 1% O_2_/94% N_2_/5% CO_2_ for 13 h, followed by reoxygenation for 6 h [19].

### 2.3. Perls' Iron Staining

Frozen slices of mouse brain were prepared as previously described [20]. Sections of different groups (*n* = 3 for each group) were stained by Perls' iron staining (Solarbio Science & Technology Co., Beijing, China). First, sections were immersed in Perls' A reagent for 30 min at room temperature and rinsed with deionized water for 5 min; then, the nuclei were stained with reagent B for 5 min, followed by rinsing the sections with deionized water for 1 minute. Finally, the sections were mounted. Statistical analysis was performed via the method described by Jiang et al. [12, 21].

### 2.4. RNA Extraction and Quantitative Real-Time PCR

Total RNA was isolated using TRIzol reagent (Invitrogen, Co., CA, USA) from the CA1 and CA3 regions of mouse hippocampus and HT22 cells, and RNA reverse transcription was performed using a commercial kit (Invitrogen Life Technologies, Carlsbad, CA, USA). Quantitative real-time PCR was conducted in triplicate on ABI 7900 Real-Time PCR System, and the relative abundance of mRNA was analyzed using the 2^-*ΔΔ*CT^ method as previously described [10]. The primers used are provided in Table 1.

### 2.5. Western Blot Analysis

The tissues of mouse hippocampus CA1 and CA3, and HT22 cells were lysed in RIPA buffer (Beyotime Institute of Biotechnology, Shanghai, China). The protein levels of TSC1 (Cell Signaling Technology, #4906), TSC2 (Cell Signaling Technology, #4308), mTOR(Cell Signaling Technology, #2983), P-mTOR (Ser2448) (Cell Signaling Technology, #5536), P-p70S6k (Thr389) (Cell Signaling Technology, #9234), and LC3A/B (Cell Signaling Technology, #4108) were determined by western blot analysis as previously described by Yang et al. [22].

### 2.6. Immunofluorescence of Tissue

The mice were sacrificed, and the brain tissue was fixed and dehydrated. Frozen sections of the mouse brain were produced for 12 *μ*m as previously reported [20]. The sections were washed with 0.01 M PBS and incubated with primary antibody and secondary antibody as described previously [20]. Rabbit anti-TSC1 (Cell Signaling Technology, Danvers, MA, USA), which was diluted 1 : 300, was incubated at 4°C overnight; the secondary antibody was diluted 1 : 500 (Jackson Laboratory, Bar Harbor, ME, USA) and incubated for 2 h at room temperature. Immunofluorescence images were examined under a laser scanning confocal microscope (A1; Nikon). The fluorescence intensity was analyzed by NIS-Element image analysis system.

### 2.7. Plasmid Construction and DNA Transfection

The TSC1-pcDNA3 plasmid was kindly provided by Dr. Nellist [3]. TSC1 was subcloned into the EGFP vector. Transfection of HT22 cells with EGFP vector, TSC1-EGFP, CRISPR-Cas9-TSC1-px330-EGFP, px330-EGFP, and GFP-LC3 plasmid was performed using Nero™ Transfection system (life technologies, Seoul, Korea) according to the manufacturer's instructions in 35 mm dishes. After transfection, cells were incubated for 24 h, and then, other experiments were carried out [12]. The primer sequences of clone and CRISPR-Cas9 used are provided in Table 2.

### 2.8. MTS Assay

HT22 cells were transfected with TSC1-EGFP and CRISPR-Cas9-TSC1-px330-EGFP plasmid by electrotransfection, and the cells were seeded in 96-well plates with DMEM. When the cells were cultured for 24 h, they grew to about 60% and were given hypoxic stimulation of 1% O_2_/94% N_2_/5% CO_2_ for 13 h and then reoxygenated for 6 h. Each well was then supplemented with 20 *μ*L/well of MTS solution (Promega Co., Madison, USA) and incubated the cells for 3 h. The absorbance at 490 nm was recorded using Multiscan FC (Thermo, MA, USA): Cell viability = (A value of treatment group − A value of zeroing hole)/(A value of control hole − A value of zeroing hole) × 100%.

### 2.9. Annexin V-FITC/PI to Detect Apoptosis

HT22 cells were transfected with TSC1-EGFP and CRISPR-Cas9-TSC1-px330-EGFP plasmid and seeded in 35 mm dishes for 24 h with hypoxic stimulation of 1% O_2_/94% N_2_/5% CO_2_ for 13 hours, followed by reoxygenation for 6 h. The cells were harvested through trypsinization and washed 3 times with cold PBS (0.15 mol/L, pH 7.2). Then, they were centrifuged at 1000 r/min for 5 min, and the supernatant was discarded. The apoptosis was detected according to the Annexin V-FITC kit (BD Co., New Jersey, USA). The cell pellet at a density of 1.0 × 10^5^-1.0 × l0^6^ cells per mL was resuspended by 1 × binding buffer. 500 *μ*L of the sample solution was transferred to a 5 mL culture tube and incubated with 5 *μ*L of FITC-conjugated annexin V and 5 *μ*L of PI for 15 min in the dark. Finally, the samples were analyzed by BD Accuri C^6^ flow cytometry: Apoptotic rate (%) = early apoptotic percentage (*Q*4) + late apoptotic percentage (*Q*2).

### 2.10. ATP Assay

When the cells were cultured for 24 h, they grew to about 60%. Then, the cells were given HPC and hypoxic stimulation following method in Section 2.2. The ATP concentration in HT22 cells was detected by an assay kit (Beyotime, Shanghai, China). The measurement of ATP concentration was performed according to the instructions of the manufacturer.

### 2.11. Reactive Oxygen Species (ROS) Assay

ROS level was detected by the assay kit (Beyotime, Shanghai, China). When HT22 cells grew to about 70%, the cells were collected in EP tubes, incubated with DCFH-DA for 20 min, and washed three times with DMEM without serum. The measurement of ROS level was performed following the instructions of the manufacturer.

### 2.12. Bisulfite-Modified DNA Sequencing (BMDS) for the DNA Methylation of CpG Sites in TSC1 Promoter Region (-720 bp~-360 bp)

Genomic DNA from hippocampal tissue was isolated using a genomic DNA extraction kit (Tiangen Co., Beijing, China) according to the manufacturer's instructions. DNA was subjected to bisulfite modification reagent using EZ DNA Methylation Glod kit (Zymo Research Co., CA, USA). The CpG island located in the promoter region (-720 bp~ -360 bp) of TSC1 gene was detected after bisulfite treatment. DNA methylation was analyzed by nested PCR; the primer sequences of it were provided in Table 3. PCR products were assessed by 1% agarose gel electrophoresis and purified by gel extraction using Zymoclean Gel DNA Recovery kit (Zymo Research Co., CA, USA). The purified products were inserted into a pEASY-T1 cloning vector (TransGen, Beijing, China) and transformed into E. coli. A minimum of 6 positive clones were chosen from LB agar plates for each sample and sequenced by the Shanghai Sangon Company.

### 2.13. Analyze the Potential Transcription Factor Binding Site

At http://bioinfo.life.hust.edu.cn/AnimalTFDB, classification and annotation of genome-wide transcription factors (TFs) as well as transcription cofactors in 97 animal genomes can be found.

### 2.14. Statistical Analysis

All data were analyzed using GraphPad Prism 8.2 and IBM SPSS version 25. Data were presented as mean ± SD. For the data of normal distribution and homogeneous variance, one-way ANOVA analysis was used and post hoc Bonferroni test was performed. When the data have normal distribution, but the variances were not homogeneous, Dunnett's T3 test was performed. A value of *P* < 0.05 was considered statistically significant.

## 3. Results

### 3.1. HPC Alleviates the Brain Injury to Hypoxia

**Figure 2:**
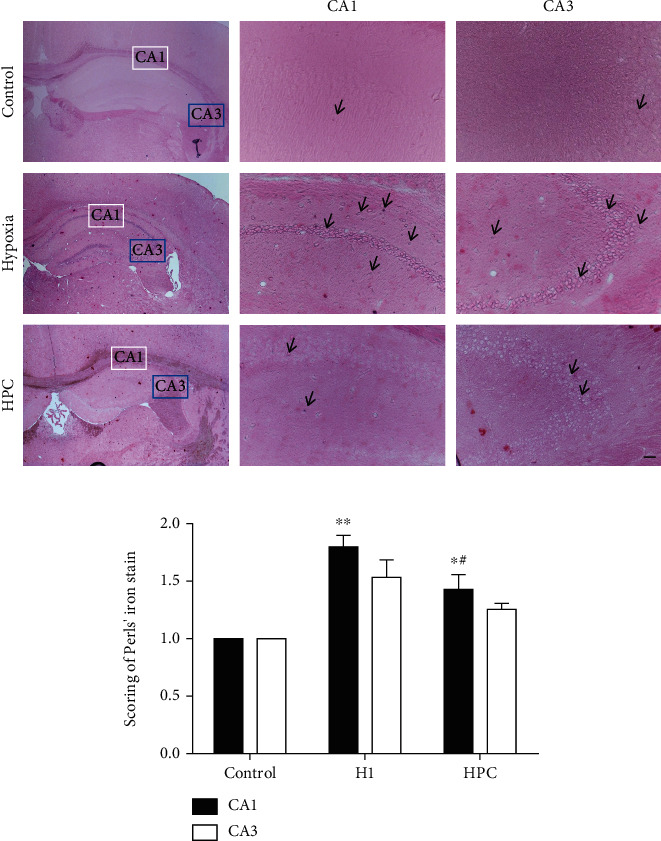
HPC alleviates the brain injury against hypoxia (*n* = 3). (a) Perls' iron stain in different groups of mouse brains. (b) The score histogram of Perls' iron stain in mouse brains (^∗∗^*P* < 0.01 vs. the control group, ^#^*P* < 0.05, ^##^*P* < 0.01 vs. the H1 group; bar = 200 *μ*m).

The injury of the brain was measured by Perls' iron staining (Figure 2(a)). There was brain injury in the H1 group with higher iron concentrations in hippocampal CA1 than in the control group (*P* < 0.01, *t* = 13.86; Figure 2(b)). Compared with that in the H1 group, the iron concentration in the CA1 region was significantly decreased in the HPC group (*P* < 0.05, *t* = 6.544; Figure 2(b)) showing the score histogram of Perls' iron staining in the mouse brain. This indicated that HPC can alleviate the injury of brain cells.

### 3.2. HPC Upregulated the Expression of TSC1 and Enhanced the Activity of Autophagy in the Hippocampal CA1 Region

**Figure 3:**
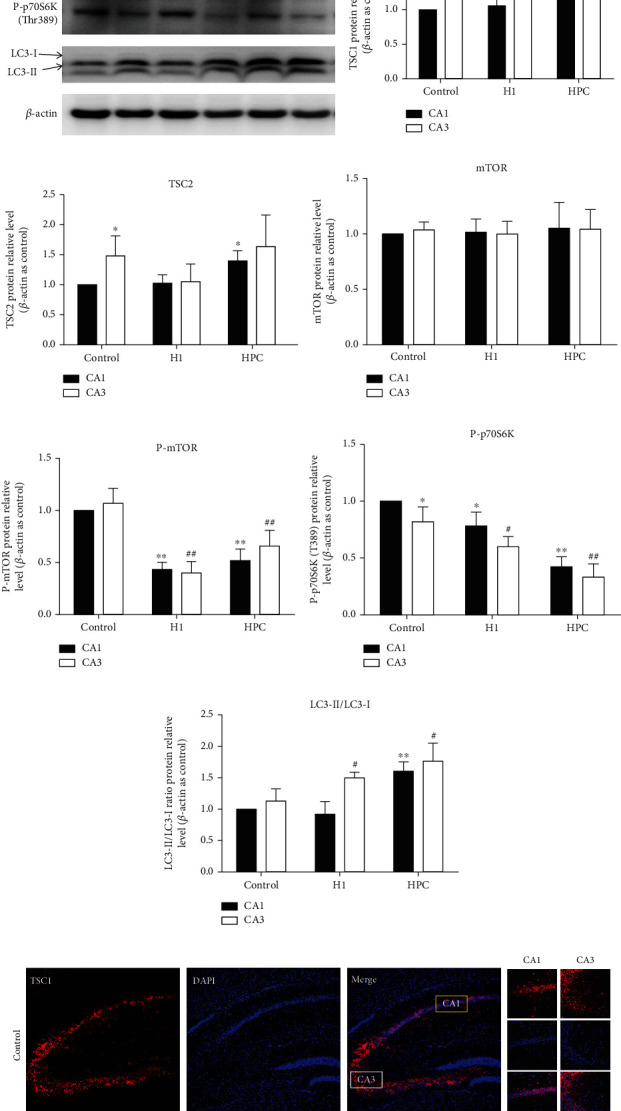
HPC upregulated the expression of TSC1 and enhanced the activity of autophagy in the hippocampal CA1 region. (a) TSC1 mRNA. (b) TSC2 mRNA. (c) mTOR mRNA. (d) LC3 mRNA. (e) Typical western blot pictures of TSC1, TSC2, mTOR, P-mTOR, P-p70S6K, and LC3-II/LC3-I ratio after exposed to HPC. (f) The relative protein levels of TSC1 in the control, H1, and HPC groups. (g) The relative protein levels of TSC2 in the control, H1, and HPC groups. (h) The relative protein levels of mTOR in the control, H1, and HPC groups. (i) The relative protein levels of P-mTOR (Ser2448) in the control, H1, and HPC groups. (j) The relative protein levels of P-p70S6K (Thr 389) in the control, H1, and HPC groups. (k) The relative protein levels of LC3-II/LC3-I ratio in the control, H1, and HPC groups. (l) The fluorescence intensity of TSC1 in hippocampus of mice. (m) The histogram of TSC1 fluorescence value analysis between the CA1 and CA3 areas in the control group, H1 group, and hypoxic preconditioning group (^∗^*P* < 0.05, ^∗∗^*P* < 0.01 vs. control CA1; ^#^*P* < 0.05, ^##^*P* < 0.01 vs. control CA3, *n* = 6 per group, bar = 200 *μ*m).

The results of real-time PCR and western blot showed that HPC could induce the upregulation of TSC1 expression in the CA1 region of the mouse hippocampus (Figures 3(a), 3(e), and 3(f)). The protein expression of TSC1 in the control group in the hippocampal CA3 area was higher than that in the hippocampal CA1 area (*P* < 0.05, *t* = 2.871, *n* = 6; Figures 3(e) and 3(f)), and the protein expression of TSC1 was upregulated by HPC in the CA1 area (*P* < 0.05, *t* = 2.598, *n* = 6; Figure 3(f)). The fluorescence intensities of TSC1 were consistent with the results of western blot (Figures 3(l) and 3(m)).

The expression of TSC2 protein was upregulated by HPC in the CA1 region, but not significantly changed in the CA3 region (*P* < 0.05, *t* = 2.444, *n* = 6; Figure 3(g)). HPC could inhibit phosphorylated mTOR (Ser2448) and phosphorylated p70S6K (Thr389) and increase the ratio of autophagy markers LC3-II/LC3-I in the CA1 and CA3 regions (*P* < 0.05; Figures 3(h)–3(k)).

### 3.3. The Methylation Changes of CpG Sites in the TSC1 Promoter Region (-720 bp~-360 bp) Were Detected by Bisulfite-Modified DNA Sequencing (BMDS)

**Figure 4:**
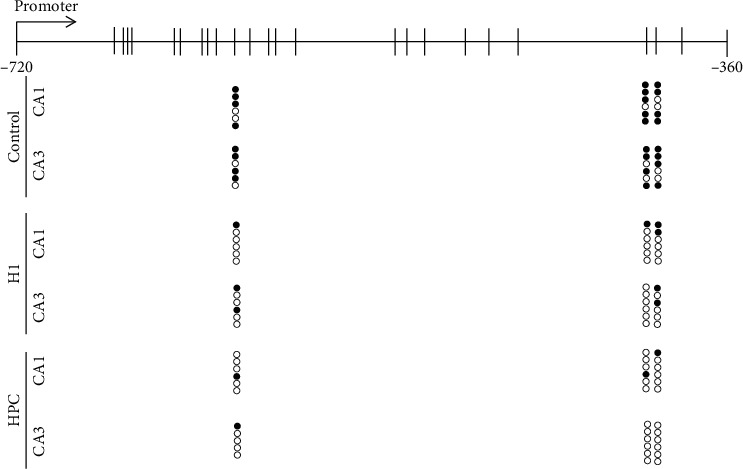
The methylation changes of CpG sites in TSC1 promoter region (-720 bp~ -360 bp) were detected by bisulfite-modified DNA sequencing (BMDS). Ten single clones are represented for each sample. The CpG island is depicted, and each circle illustrates a single CpG. Closed circles represent methylated CpG, whereas open circles represent unmethylated CpG.

The BMDS results showed that there was no significant change in the overall methylation level of CpG sites in the promoter (-720 bp~ -360 bp) region of TSC1 in the control group, hypoxic group, and HPC group. It was worth noting that the 10^th^, 21^st^, and 22^nd^ sites of CpG demethylation were changed after hypoxia and HPC treatment in CA1 and CA3. The methylation rates at the 10^th^, 21^st^, and 22^nd^ sites were 66.67%, 83.33%, and 66.67% (control); 16.67%, 16.67%, and 33.33% (H1 group); 16.67%, 16.67%, and 16.67% (HPC group) in CA1, respectively. In the CA3 region of the mouse hippocampus, the methylation rates at the 10th, 21^st^, and 22^nd^ sites were 66.67%, 66.67%, and 66.67% (control); 33.33%, 0%, and 33.33% (H1 group); 16.67%, 0%, and 0% (HPC group), respectively (Figure 4).

Online software animal TFDB (http://bioinfo.life.hust.edu.cn/AnimalTFDB) was used to analyze the potential transcription factors (TFs) for the three sites. The analysis revealed that there were more than 100 transcription factors that can bind to the sequence of the 10^th^ CpG site in the promoter (such as EP300 and SP3), 3 transcription factors (NFIC, ZKSCAN1, and ZBTB17) and 2 transcription factors (ZKSCAN1 and ZBTB17) that can bind with the sequence of the 21^st^ and 22^nd^ CpG site, respectively.

### 3.4. HPC Induced the TSC1 Expression and the Activity of mTOR/p70S6K/Autophagy Pathway with the Change of ATP Consumption and ROS Production in HT22 Cells

**Figure 5:**
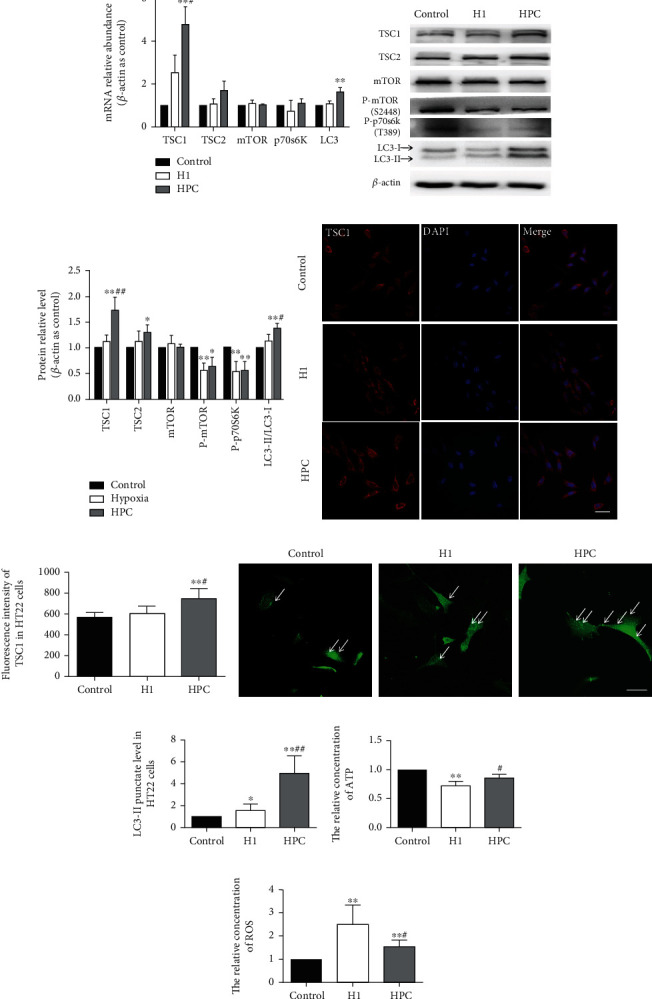
HPC induced the TSC1 expression and the activity of mTOR/p70S6K/autophagy pathway with the change of ATP consumption and ROS production in HT22 cells. (a) The relative mRNA abundance of TSC1, TSC2, mTOR, p70S6K, and LC3. (b) Typical western blot pictures of TSC1, TSC2, mTOR, P-mTOR (Ser2448), P-p70S6K (Thr389), and LC3-II/LC3-I ratio protein levels in HT22 cells. (c) The relative protein levels of TSC1, TSC2, mTOR, P-mTOR (Ser2448), P-p70S6K (Thr389), and LC3-II/LC3-I ratio in the control, H1, and HPC groups. (d) The fluorescence intensity of TSC1 in HT22 cells in the control group, H1 group, and HPC group. (e) The histogram of TSC1 fluorescence value analysis in the control, H1, and HPC groups. (f) The pictures of pe-GFP-LC3-II in the control, H1, and HPC groups. (g) The histogram of pe-GFP-LC3-II in the control, H1, and HPC groups. (h) Relative concentration of ATP in the control, H1, and HPC groups of HT22 cells. (i) Relative concentration of ROS in the control, H1, and HPC groups of HT22 cells (^∗^*P* < 0.05, ^∗∗^*P* < 0.01 vs. the control group, ^#^*P* < 0.05 vs. the H1 group, *n* = 6 per group, bar = 50 *μ*m).

To further confirm the effects of TSC1 and TSC complex downstream on neuroprotection in vivo after HPC, the changes were detected by an HPC in vitro model. The expression of TSC1 was statistically increased in both mRNA and protein levels in the HPC group (*P* < 0.05, Figures 5(a)–5(c)). In morphology, the results of immunofluorescence showed that the TSC1 protein was mainly expressed in the cytoplasm of HT22 cells, and the fluorescence intensity of TSC1 protein in the HPC group was stronger than that in the control group and H1 group. The result of immunofluorescence suggested that the expression of TSC1 was increased (Figures 5(d) and 5(e)). The results were consistent with western blot results.

HPC upregulated TSC2 protein level and did not affect its mRNA level (Figures 5(a)–5(c)). At the same time, the levels of phosphorylated mTOR (Ser2448) and phosphorylated p70S6K (Thr389) proteins were found to decrease in the HPC and H1 groups (*P* < 0.05, Figures 5(b) and 5(c)). The mRNA expression of LC3 and LC3-II/LC3-I ratio protein were increased in the HPC group (*P* < 0.05, Figures 5(a)–5(c)). The puncta of LC3II, indicating autophagy, was significantly increased in HT22 cells transfected with GFP-LC3 in the HPC group compared with that in the control and H1 group (*P* < 0.05, Figures 5(f) and 5(g)). The results showed that the autophagy activity was induced by HPC.

The results of ATP assay indicated that ATP concentration in the hypoxic group was significantly decreased compared with that in the control group (*P* < 0.01, *t* = 7.920, *n* = 6; Figure 5(h)). HPC can restore ATP concentration compared to the H1 group (*P* < 0.05, *t* = 2.895, *n* = 6; Figure 5(h)). The ROS level was increased in the H1 group compared with that in the control group (*P* < 0.05, *t* = 4.009, *n* = 6; Figure 5(i)), and the ROS could be reduced in the HPC group compared with that in the H1 group (*P* < 0.05, *t* = 2.426, *n* = 6; Figure 5(i)).

### 3.5. TSC1 Contributes to Cell Viability and Apoptosis, Which Is Related to ATP Consumption and ROS Production under Hypoxia Conditions in HT22 Cells

**Figure 6:**
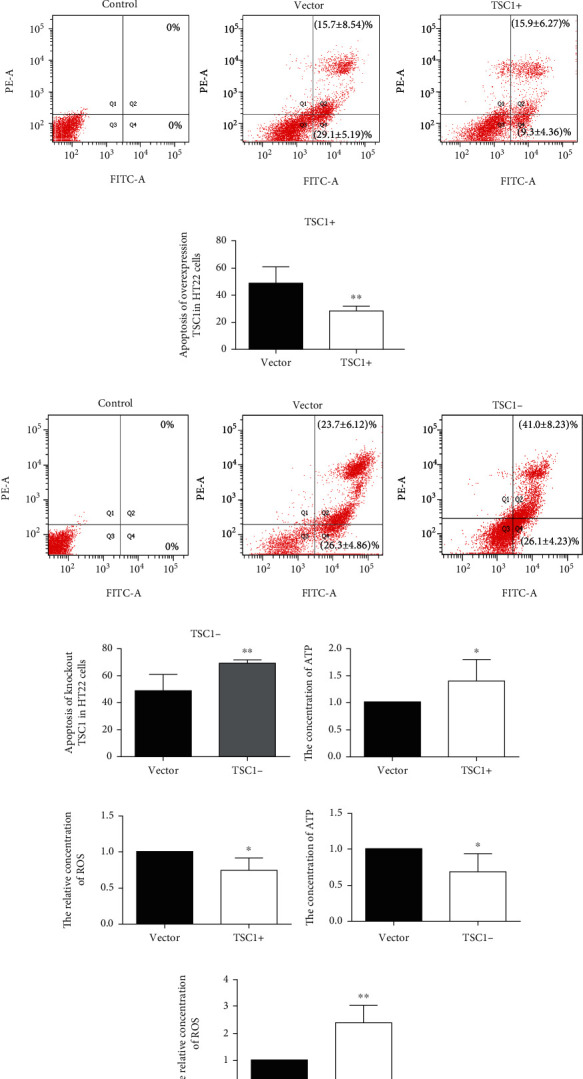
TSC1 contributes to cell viability and apoptosis, which is related to ATP consumption and ROS production under hypoxia conditions in HT22 cells. (a) Overexpression of the TSC1 gene could increase the cell viability in HT22 cells when exposed to hypoxia. (b) Knockout of TSC1 gene in HT22 cells could decrease the cell viability in HT22 cells when exposed to hypoxia. (c) Overexpression of the TSC1 gene could reduce the cell apoptosis in HT22 cells when exposed to hypoxia. (d) The histogram of apoptosis of overexpressed TSC1. (e) Knockout of the TSC1 gene could increase the cell apoptosis in HT22 cells when exposed to hypoxia. (f) The histogram of apoptosis of knockdown TSC1. (g) Overexpressed TSC1 gene upregulated ATP concentration in HT22 cells when exposed to hypoxia. (h) Overexpressed TSC1 gene could decrease ROS concentration in HT22 cells when exposed to hypoxia. (i) Knockout of TSC1 gene could downregulate ATP concentration in HT22 cells when exposed to hypoxia in HT22 cells. (j) Knockout of TSC1 gene could increase ROS concentration in HT22 cells when exposed to hypoxia in HT22 cells (^∗^*P* < 0.05, ^∗∗^*P* < 0.01 vs. the vector group).

TSC1 was overexpressed and knocked down in HT22 cell, which was electrotransfected with TSC1-EGFP and CRISPR-Cas9-TSC1-px330-EGFP plasmids, respectively. The cell viability was detected by MTS, and cell apoptosis was measured by flow cytometry after hypoxic treatment. The cell viability increased, and cell apoptosis decreased in HT22 cells, which overexpressed TSC1 (*P* < 0.01, *t* = 13.93, 3.282; Figures 6(a) and 6(c)). On the contrary, the cell viability decreased and cell apoptosis increased in HT22 cells with knockdown of TSC1 (*P* < 0.05, *t* = 15.24, 2.807; Figures 6(b) and 6(e)).

HT22 cells, which overexpressed the TSC1 gene, showed higher ATP and lower ROS levels compared to the vector group (*P* < 0.05, *t* = 2.354, 2.944, *n* = 6; Figures 6(g) and 6(h)). On the contrary, HT22 cells with knockdown of TSC1 displayed lower ATP levels and higher ROS compared to the vector group (*P* < 0.05, *t* = 3.124, 4.452, *n* = 6; Figures 6(i) and 6(j)).

The results implied that TSC1 contributes to neuroprotection by regulating metabolism and reducing ROS production under hypoxic conditions.

## 4. Discussions

The differential vulnerability of CA1 and CA3 neurons to ischemia has been studied extensively [23, 24]. The “neuronal factors” underlying the differential vulnerability of CA1 versus CA3 have been of great interest [6]. The genes of “neuronal factors” should be differentially expressed under normoxic and ischemic conditions in CA1 and CA3 [24]. Until now, it is unclear why TSC1 is higher in CA3 and why it can be induced by IPC in CA1. DNA methylation of TSC1 affects its expression [25]. In the current study, we explored the possible role of DNA methylation in TSC1 expression and its role in the differential susceptibility of CA1 and CA3 neural cells under hypoxia conditions.

Mammalian brain cells show remarkable diversity in gene expression, anatomy, and function, which is associated with DNA methylation dynamics [26]. In humans, the low level of DNA methylation in the promoter region or the lack of “open” configuration of chromatin is related to the interaction between DNA and the transcriptional complexes, which leads to the activation of gene expression. In contrast, methylation of CpG islands in gene promoters is associated with the “off” configuration of chromatin that causes gene silencing [27]. Hartley et al. reported that the expression levels of some genes may be related to the changes of their DNA methylation, and they used RNA-Seq to measure neuronal cells under hypoxic stress at 1% O_2_ and found that CpG islands tended to maintain hypomethylation for a long time after eliminating the hypoxia stress [28]. Wang et al. found that the decreased expression of TSC1 was related to the promoter methylation status of TSC1 [29]. We had reported that decreasing the DNA methylation level of TSC1 promoter by DNMT inhibitors can increase its expression [12]. Zhang et al. showed that food-induced TSC1 promoter hypermethylation in rat hypothalamic sites leads to decreased TSC1 expression [25]. HPC did not change the total DNA methylation level of the TSC1 promoter fragment (-720 bp to -360 bp) in this study, whereas HPC downregulated the three CpG sites (10, 21, 22) in this region with the increase of TSC1 expression in both CA1 and CA3. Since we only checked a small fragment and did not check other regions of the promoter of TSC1, we are still not very sure that the change in TSC1 expression correlates with its promoter DNA methylation. The three CpG sites can bind to transcription factor (TF) as predicted by bioinformation. Therefore, we proposed that DNA methylation and TF may contribute to the expression of TSC1 and this proposal needs to be tested further.

The upregulation of TSC1 inhibits the mTOR pathway by reducing the energy demand of cells, thereby providing protection against hypoxic/ischemic injury. A commonality between the mechanisms that underlie the HPC and IPC is the metabolic depression of cerebral metabolism and activity to produce a hibernation-like state, the ability to regulate body temperature and thus metabolic rate lies at the center of metabolic depression [1]. We found that HPC can induce TSC1 expression in both mRNA and protein levels in CA1. Our results are similar to those reported by Papadakis [7]. In addition, we found that both TSC1 and TSC2 protein levels were higher in CA1 than that in CA3 under normoxia conditions, and TSC1 can be upregulated in CA3, whereas it was unchanged in CA1 after hypoxic treatment. It is well known that the TSC complex can inhibit the mTOR pathway, which is critical for controlling cell energy metabolism in neural cells [30]. The mTOR pathway activity of P-mTOR and P-p70S6k protein levels was opposite to the level of TSC1 in CA1 and CA3 in the current study [12]. Yang et al. reported that inhibition of mTOR by rapamycin markedly reduced ischemia-induced brain damage [31]. Hwang et al. found that CA1 P-mTOR was downregulated after global cerebral ischemia, and autophagy was upregulated to protect CA1 neurons [32]. The increased autophagy level in CA1 of HPC and in CA3 of both H and HPC may reduce neuronal cell injury in this study. Moderate autophagy is a well-known physiological process that prolongs cell survival through the recycling of cellular macromolecules to generate energy and is beneficial to the neuronal cell under stress [33]. Therefore, the differential expression of TSC1 in CA1 and CA3 under hypoxia may moderately upregulate autophagy through mTOR activity, to promote hypoxia/ischemia tolerance, which depends on the changes of energy metabolism.

HT22 cells were used as an in vitro system to further validate the effect of HPC on TSC1 expression and the role of TSC1 in energy metabolism. HT22 cells treated with HPC can increase cell viability and inhibit cell apoptosis [19]. The changes of TSC1 and mTOR pathway/autophagy in HPC HT22 showed a similar pattern in CA1 of HPC mice, while HPC can restore the ATP concentration compared to hypoxia. The hippocampal neuron oxygen-glucose deprivation (OGD) experiment also proved that the reduction in mTOR and the increase in autophagy markers play a protective role [32]. At the same time, our data showed that overexpression and knockdown of TSC1 can increase ATP concentration and decrease ROS concentration in HT22 cells after hypoxia/reoxygenation, respectively. The key role of TSC1 in modulating metabolic has been shown in TSC1-deficient dendritic cells, which exhibit increased glycolysis and mitochondrial respiration [34]. Choo and colleagues found that TSC1/2*^−/−^* cells were hypersensitive to glucose deprivation, which was associated with the activation of apoptosis due to the decrease of ATP levels [15].

**Figure 7:**
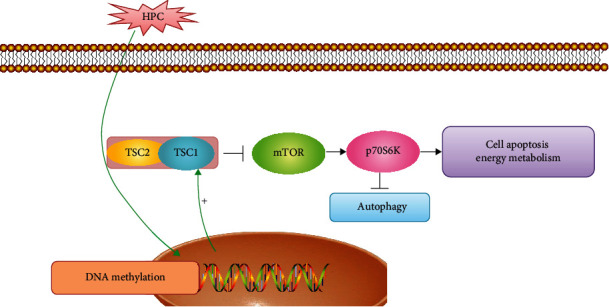
The proposed neuroprotective function of TSC1 induced by HPC.

In conclusion, TSC1 can be induced by HPC, which may be related to the methylation level of its promoter, and the neuroprotection of TSC1 may affect energy metabolism through the mTOR/autophagy pathway. And this possible mechanism was shown in Figure 7. At the same time, our data supported that TSC1 expression is involved in the differential vulnerability of CA1 and CA3 neurons. We only measured a DNA fragment of TSC1 promoter (-720 bp~ -360 bp) and 3 sites were changed after hypoxia treatment. The relationship between TSC1 DNA methylation level and its expression under hypoxic/ischemic conditions needs to be further investigated in the future.

## Figures and Tables

**Table 1:** Primer sequence.

Primer	Forward 5′ →3′	Reverse 5′ →3′
TSC1	ATGGCCCAGTTAGCCAACATT	CAGAATTGAGGGACTCCTTGAAG
mTOR	CAGTTCGCCAGTGGACTGAAG	GCTGGTCATAGAAGCGAGTAGAC
LC-3	TTATAGAGCGATACAAGGGGGAG	CGCCGTCTGATTATCTTGATGAG
*β*-Actin	AGGTGAAGGTCGGAGTCA	GGTCATTGATGGCAACAA

**Table 2:** Primer sequences of clone and CRISPR-Cas9.

Primer	Forward 5′ →3′	Reverse 5′ →3′
TSC1-EGFP-N1	TGCAGCTAGCATGGCCCAACAAGCAAAT	CTAGGGTACCATGCTGTGTTCATGATGAGT
CRISPR-Cas9-TSC1-px330-EGFP	CACCGTCTTTGGCCGTCTCTCGTCA	CAGAAACCGGCAGAGAGCAGTCAAA

**Table 3:** Primer sequences of nested PCR.

Primer	Forward 5′ →3′	Reverse 5′ →3′
TSC1-out	AGAGGTTTATTTGGAAATAGGAA	ACGCGCAACACCTCCCCCT
TSC1-in	AGATTTTAAAGGTGGTAGGTGTG	CTATAAAAAAAAATCCCTATT

## Data Availability

The tables and figures used to support the findings of this study are included within the article.

## Supplementary Materials

Supplementary Materials Expression of TSC1 upon overexpression or knockout of TSC1 gene in HT22 cells.
