# Supplementary material for: Possible Involvement of DNA Methylation in TSC1 Gene Expression in Neuroprotection Induced by Hypoxic Preconditioning
Source: Oxid Med Cell Longev. 2022 Sep 9;2022:9306097. doi: 10.1155/2022/9306097 (PMC9481362; doi:10.1155/2022/9306097)
Supplement: Supplementary Materials — Expression of TSC1 upon overexpression or knockout of TSC1 gene in HT22 cells. [file 9306097.f1.docx]

**Supplementary Materials**

**
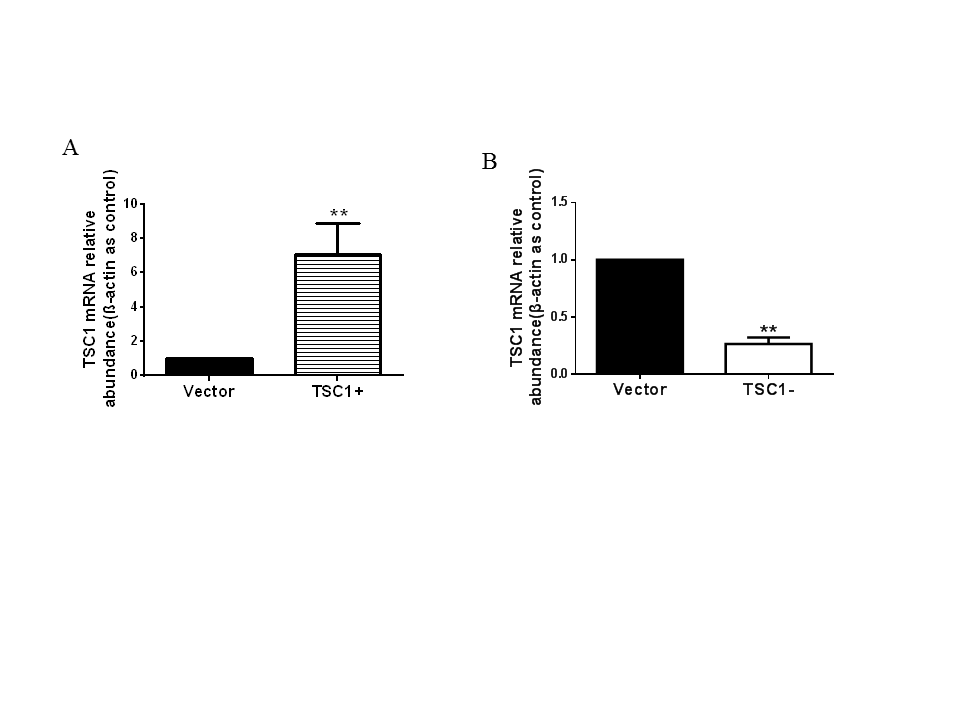
**

**
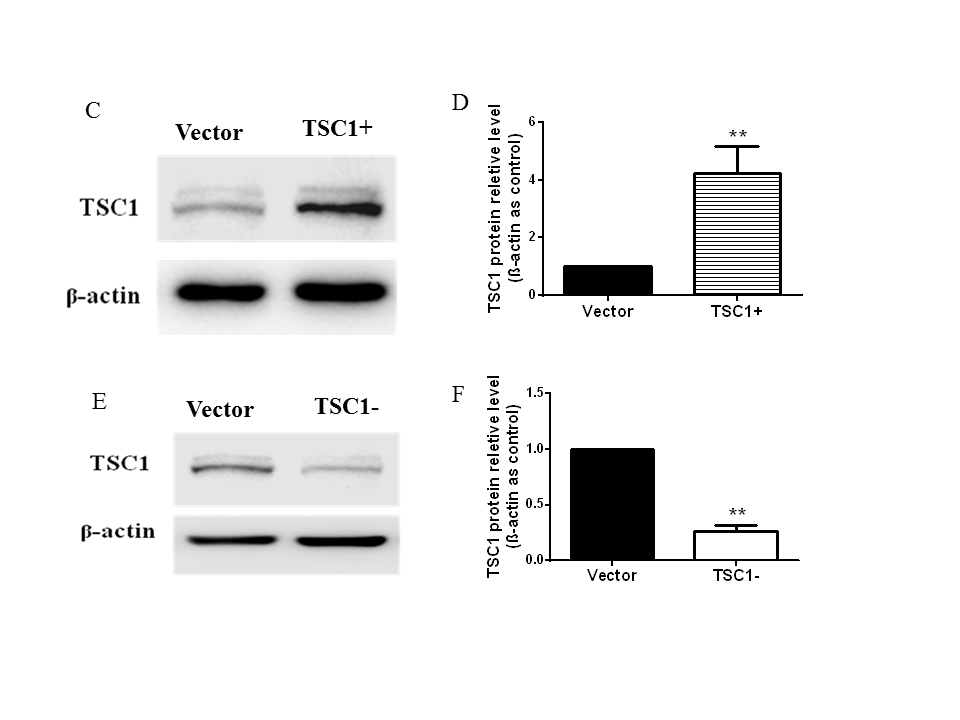
**

**Figure S1.** Expression of TSC1 upon over-expression or knockout of TSC1 gene in HT22 cells.

A: The relative mRNA abundance of TSC1 upon over-expression of TSC1; B: The relative mRNA abundance of TSC1 upon knockout of TSC1; C: Typical Western Blot pictures of TSC1 protein upon over-expression of TSC1; D: The relative protein levels of TSC1 in vector and over-expression TSC1 group; E: Typical Western Blot pictures of TSC1 protein upon knockout of TSC1; F: The relative protein levels of TSC1 in vector and knockdown TSC1 group.

(**<0.01*vs* vector group, n=3 per group).
